# Pathological Role of Phosphoglycerate Kinase 1 in Balloon Angioplasty-Induced Neointima Formation

**DOI:** 10.3390/ijms22168822

**Published:** 2021-08-17

**Authors:** Chun-Hsu Pan, Yi-Chung Chien, Min-Shan Sung, Hui-Yu Huang, Ming-Jyh Sheu, Chieh-Hsi Wu

**Affiliations:** 1 Ph.D. Program in Drug Discovery and Development Industry, College of Pharmacy, Taipei Medical University, Taipei 11031, Taiwan; panch@tmu.edu.tw; 2 School of Pharmacy, Taipei Medical University, Taipei 11031, Taiwan; 3 Drug Development Center, China Medical University, Taichung 40402, Taiwan; hardway19800710@gmail.com; 4 Center for Molecular Medicine, China Medical University Hospital, Taichung 404332, Taiwan; 5 Graduate Institute of Basic Medical Science, China Medical University, Taichung 40402, Taiwan; ann@allbio.com.tw; 6 Graduate Institute of Metabolism and Obesity Sciences, Taipei Medical University, Taipei 11031, Taiwan; maggieh323@hotmail.com; 7 School of Pharmacy, China Medical University, Taichung 40402, Taiwan; soybean13mtdtw@gmail.com

**Keywords:** hypoxia, neointimal formation, phosphoglycerate kinase 1, platelet-derived growth factor receptor-β, vascular smooth muscle cells

## Abstract

Restenosis is a common vascular complication after balloon angioplasty. Catheter balloon inflation-induced transient ischemia (hypoxia) of local arterial tissues plays a pathological role in neointima formation. Phosphoglycerate kinase 1 (PGK1), an adenosine triphosphate (ATP)-generating glycolytic enzyme, has been reported to associate with cell survival and can be triggered under hypoxia. The purposes of this study were to investigate the possible role and regulation of PGK1 in vascular smooth muscle cells (VSMCs) and balloon-injured arteries under hypoxia. Neointimal hyperplasia was induced by a rat carotid artery injury model. The cellular functions and regulatory mechanisms of PGK1 in VSMCs were investigated using small interfering RNAs (siRNAs), chemical inhibitors, or anaerobic cultivation. Our data indicated that protein expression of PGK1 can be rapidly induced at a very early stage after balloon angioplasty, and the silencing PGK1-induced low cellular energy circumstance resulted in the suppressions of VSMC proliferation and migration. Moreover, the experimental results demonstrated that blockage of PDGF receptor-β (PDGFRB) or its downstream pathway, the phosphoinositide 3-kinase (PI3K)-AKT-mammalian target of rapamycin (mTOR) axis, effectively reduced hypoxia-induced factor-1 (HIF-1α) and PGK1 expressions in VSMCs. In vivo study evidenced that PGK1 knockdown significantly reduced neointima hyperplasia. PGK1 was expressed at the early stage of neointimal formation, and suppressing PGK1 has a potential beneficial effect for preventing restenosis.

## 1. Introduction

Percutaneous transluminal coronary angioplasty (PTCA)-caused mechanical injury in arterial walls triggers pathological adaptive responses that lead to recurrence of stenosis (restenosis). This response is initiated by arterial deendothelialization, resulting in platelets adhered and activated on the lumen surface of injured arteries as well as released mitogens, such as vascular endothelial growth factor (VEGF) and platelet-derived growth factor (PDGF), to facilitate cell proliferation and migration of vascular smooth muscle cells (VSMCs) [1,2,3]. Therefore, abnormal proliferation and migration of VSMC within the vascular media layer have been believed to be the major factors resulting in balloon-injured artery restenosis [4,5,6]. As the progression of restenosis begins immediately after angioplasty injury, elucidation of the molecular changes at the early stages of restenosis progression will help us to discover potential novel therapeutic targets for the prevention of restenosis.

Arterial ischemia-reperfusion injury occurred by catheter balloon inflation-caused obstruction in blood flow during the surgical process of PCTA. Under the hypoxic situations, an oxygen-sensitive transcription factor, hypoxia-inducible factor-1 (HIF-1), was quickly induced to play a critical role in regulating cell survival, and was thus involved in restenosis progression [7,8,9]. Suppressing local expression of HIF-1 can effectively reduce arterial injury-induced neointima formation [9], suggesting hypoxia plays a role in regulatory mechanisms in restenosis progression. Phosphoglycerate kinase 1 (PGK1), a glycolytic enzyme, produces the first ATP molecule in glycolysis by catalyzing the conversion of 1,3-diphosphoglycerate to 3-phosphoglycerate. PGK1 is also a HIF-1-targeted gene, which can facilitate catabolism and energy production required for cell survival and proliferation [10,11,12]. PGK1 inhibitors have been shown to induce cell apoptosis and to inhibit tumor cell adhesion and metastasis [13,14,15], whose pharmacological effects implied that PGK1 may have similar regulations in VSMC proliferation and migration. In addition, some growth factors, such as VEGF and PDGF, were found to be upregulated by HIF-1 [16,17]. Both PDGF and VEGF have been demonstrated as restenosis-promoting growth factors that can facilitate the development of restenosis [18,19]. To date, it remains unclear whether there are interactions that exist among PDGF, VEGF, and PGK1 under hypoxic condition. Accordingly, the aims of this study were to understand the expression profile of PGK1 in balloon inflation-injured arterial walls during neointimal formation, to investigate the pathological role of PGK1 in VSMC proliferation and migration, to clarify the effects of potential growth factors on PGK1 expression in VSMCs, and to evaluate whether suppressing PGK1 has a beneficial effect for reducing balloon injury-induced neointimal hyperplasia.

## 2. Results

### 2.1. Protein Level of PGK1 Was Rapidly Elevated in Arteries after Balloon Angioplasty

**Figure 1:**
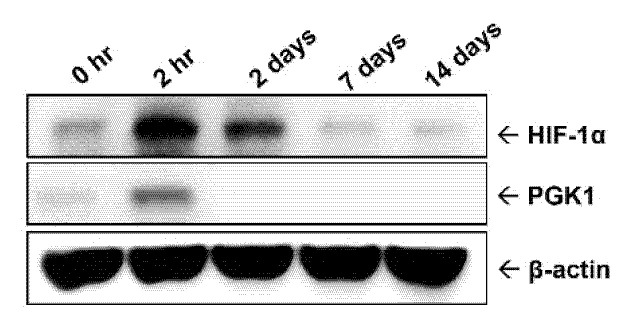
Temporal expression profiles of HIF-1α and PGK1 proteins in balloon-injured arteries.

For understanding ischemic (or hypoxic) status in balloon-injured arteries, the arteries were respectively collected at different time points (0 h, 2 h, 2 days, 7 days, and 14 days) over a 14-day period following balloon angioplasty. Hypoxic status was evaluated by detecting intratissual level of HIF-1α protein, a well-known hypoxia marker. The result of intra-arterial hypoxic status showed that HIF-1α protein was rapidly increased at 2 h after balloon injury and then gradually declined at 2 days post-surgery (Figure 1). Similarly, the protein level of PGK1 was transiently up-regulated at 2 h post-injury and rapidly returned to basal level at 2 days in the injured lesions.

### 2.2. PGK1 Knockdown Resulted in Decreases in VSMC Proliferation and Migration

**Figure 2:**
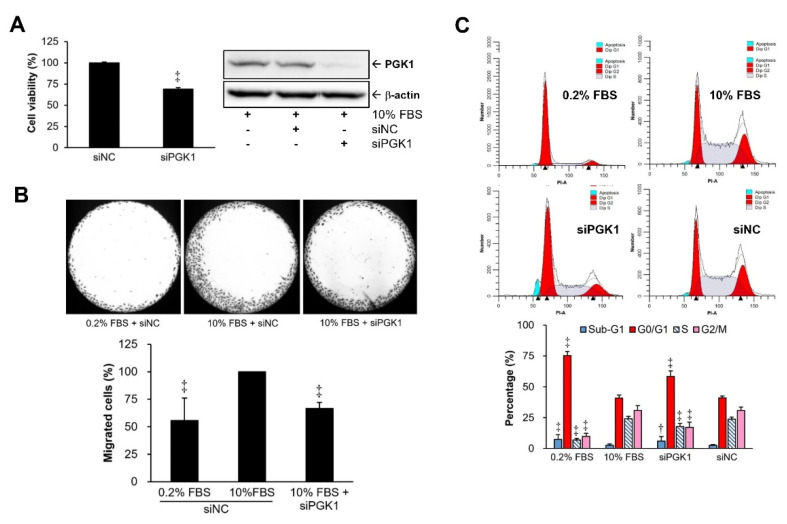
Potential roles of PGK1 in VSMC growth and migration. A10 cells with 24 h siRNA transfection were further used to examine cell viability, and silencing efficiency of PGK1 siRNA (siPGK1) was confirmed by immunoblot (**A**). An Oris^®^ cell migration assay kit was used to evaluate cell migration in VSMCs with PGK1 knockdown (**B**). The influence of silencing PGK1 on distribution of cell-cycle phases was also analyzed using a flow cytometer (**C**). siNC, the negative control (scramble) siRNA. All siRNAs were applied on VSMCs under culture medium with 10% (*v*/*v*) fetal bovine serum (FBS). Photographs of each group were taken at 40× magnifications. Data are representative of at least three independent experiments (*n* = 6/group). † *p* < 0.05 and ‡ *p* < 0.01 compared with the control group (10% FBS + siNC).

PGK1 gene was silenced by specific siRNA in VSMCs to investigate whether PGK1 plays regulatory roles in VSMC proliferation and migration. Our data showed that cell proliferation (Figure 2A) and migration (Figure 2B) could be significantly suppressed after PGK1 silencing in VSMCs. PGK1 siRNA (siPGK1) provided more than 80% silencing efficiency at protein level as compared with the negative control group (siNC). Besides, our data also demonstrated that PGK1 knockdown-induced growth suppression via increases of G1 (cell-cycle arrest) and sub-G1 (apoptosis) phases could be found in the siPGK1 group compared with the siNC group (Figure 2C).

### 2.3. Silencing PGK1 Increased Intracellular Adenosine Diphosphate (ADP)/ATP Ratio to Trigger Activation of Adenosine 5’-Monophosphate (AMP)-Activated Protein Kinase (AMPK) 

**Figure 3:**
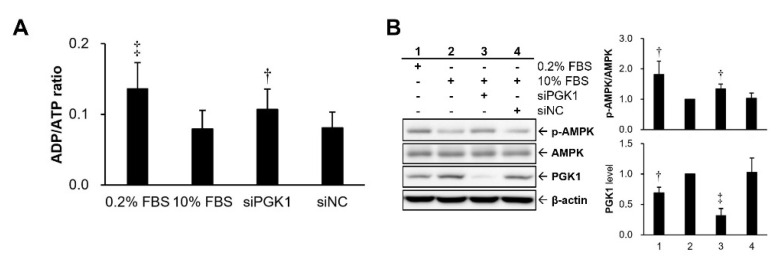
Effect of silencing PGK1 in the intracellular energy status of VSMCs. A10 cells with 24 h siRNA transfection were further used to examine intracellular energy homeostasis (**A**) and the activation status of AMPK protein (**B**). siNC, the negative control (scramble) siRNA. All siRNAs were applied on VSMCs under 10% FBS culture medium. Data are representative of at least three independent experiments (*n* = 6/group). † *p* < 0.05 and ‡ *p* < 0.01 compared with the control group (10% FBS + siNC).

To further clarify whether growth suppression by PGK1 silencing resulted from an imbalance of intracellular energy status, an index of cellular energy status, cytosolic ADP/ATP ratio, was further examined in VSMCs after PGK1 silencing. A higher ADP/ATP ratio means the cells stayed at a lower energy state. Our data revealed that the ADP/ATP ratio was increased in VSMCs with knockdown of PGK1 as compared with that of the group transfected with siNC or 10% FBS group (Figure 3A). For double confirming the cellular energy status, the activation level of AMPK protein, a cellular energy sensor, was measured. The experimental results indicated that PGK1 knockdown significantly elevated the expression level of activated AMPK protein as compared with that of the siNC group (Figure 3B).

### 2.4. Suppressing PGK1 Can Improve Balloon Injury-Induced Neointimal Formation

**Figure 4:**
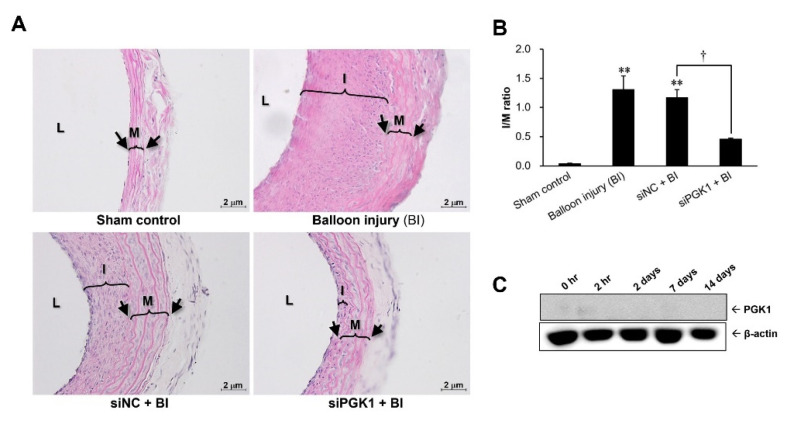
Influence of PGK1 knockdown in balloon injury-induced neointima formation. Neointima hyperplasia of the carotid arteries was induced by balloon angioplasty, and intra-arterial PGK1 expression was knocked down by siRNA intervention at the same time. Carotid arteries were harvested two weeks after balloon angioplasty to examine histopathological changes of arterial tissues after silencing PGK1 (**A**). Black arrow indicates locations of the internal and external elastic fiber, respectively. L, lumen; I, (neo)intima layer; M, media layer; siNC, negative control siRNA; siPGK1, PGK1 siRNA. The manifestation of neointimal hyperplasia was presented as the ratio of (neo)intima-to media area (I/M ratio) (*n* = 3 mice/group) (**B**). The silencing efficiency of PGK1 siRNA was confirmed using immunoblot to examine the intra-arterial level of PGK1 protein (**C**). ** *p* < 0.01 compared with the sham control. † *p* < 0.05 compared with the siNC + BI group.

For evaluating whether PGK1 could be used as a therapeutic target to prevent neointima hyperplasia, siPGK1 was applied in a rat carotid artery balloon injury model. The severity of neointima hyperplasia was evaluated according to the ratio of (neo)intima-to-media area (I/M ratio). As shown in Figure 4A, at 14 days post-injury, the thickening of the intimal layer was apparent in the carotid artery of both the balloon injury (BI) and the siNC + BI groups as compared with the sham control group. In contrast, the thickness of the intimal layer was markedly reduced in the carotid artery from the siPGK1 + BI group. These results were confirmed by the quantitative measurement of the I/M ratio in the carotid arteries (Figure 4B). The I/M ratios in both the BI and the siNC + BI groups had a marked increase to about 1.2~1.3, as compared with ~0.04 in the sham control group, but the I/M ratio in the carotid artery transfected with siPGK1 increased only to 0.4~0.5. For checking the in vivo silencing efficiency of siPGK1, the expression level of PGK1 protein was detected in the siPGK1-transfected injured arteries harvested from different time points after surgery. Our data revealed that in vivo delivery of siPGK1 can obviously abolish the protein expression of PGK1, especially at 2 h post-surgery (Figure 4C).

### 2.5. Hypoxia and PDGF-BB Can Be Served as Stimulatory Factors for PGK1 Expression in VSMCs

**Figure 5:**
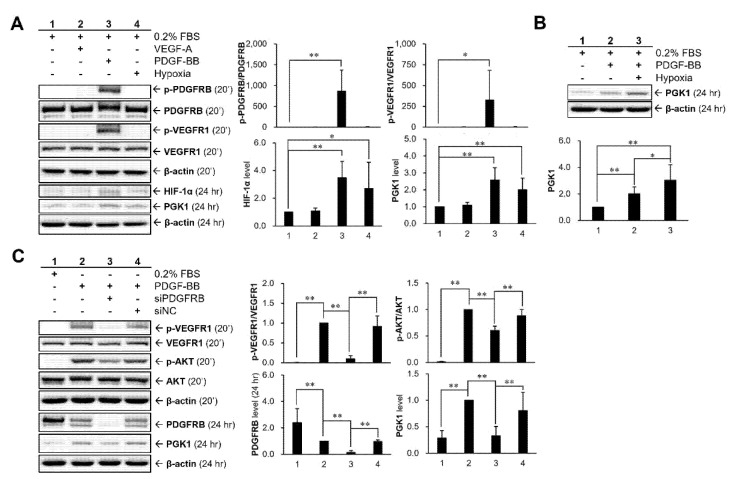
Regulatory effects of hypoxia and growth factors (VEGF-A and PDGF-BB) on PGK1 expression in VSMCs. Cells were treated with 25 ng/mL of recombinant proteins (VEGF-A and PDGF-BB) or with the hypoxic condition for 24 h under low-serum (0.2% FBS) medium (**A**). Synergistic effects of hypoxia and PDGF-BB on PGK1 expression were investigated under the low-serum condition (**B**). Investigation of the role of VEGF receptor-1 (VEGFR1) on PDGF-BB-mediated PGK1 up-regulation (**C**). siPDGFRB, PDGFRB siRNA; siNC, negative control siRNA. Data are representative of three independent experiments. The significance level is denoted with * for *p* < 0.05 and ** for *p* < 0.01.

For understanding whether hypoxia and some restenosis-associated molecules (VEGF and PDGF) were involved in PGK1 expression in VSMCs, the cells were treated with 25 ng/mL of recombinant proteins (VEGF-A and PDGF-BB) or with hypoxic condition for 24 h under low-serum (0.2% FBS) medium (Figure 5A). Our results showed that PDGF-BB stimulation and hypoxic condition can markedly induce the expressions of PGK1 and HIF-1α proteins. These data also found that VEGF receptor-1 (VEGFR1) and PDGF receptor-beta (PDGFRB) could be activated (phosphorylated) by PDGF-BB. Moreover, cells were treated with PDGF-BB under hypoxic condition to investigate whether the combination treatment (PDGF-BB + hypoxia) could produce a synergistic (or additive) effect in regulating the expression of PGK1 protein (Figure 5B). Based on the analyzed result, the hypoxic condition did have a significant effect to enhance PDGF-BB-induced PGK1 expression under the experimental condition. Moreover, for further investigating whether or not PDGF-BB-induced VEGFR1 activation is a PDGFRB-dependent regulation, PDGFRB siRNA (siPDGFRB) was applied to clarify the relationship between PDGF-BB and VEGFR1 activation (Figure 5C). Our data revealed that PDGF-BB-induced VEGFR1 activation is a PDGFRB-dependent regulation rather than a direct interaction between PDGF-BB and VEGFR1. Interestingly, the activation level of AKT, a downstream molecule of PDGFRB, was not fully abolished after PDGFRB silencing, which implied that PDGF-BB-mediated VEGFR1 activation may contribute partly to the PDGF-BB-triggered AKT activation.

### 2.6. HIF-1α Participated in PDGF-BB-Mediated PGK1 Expression in VSMCs

**Figure 6:**
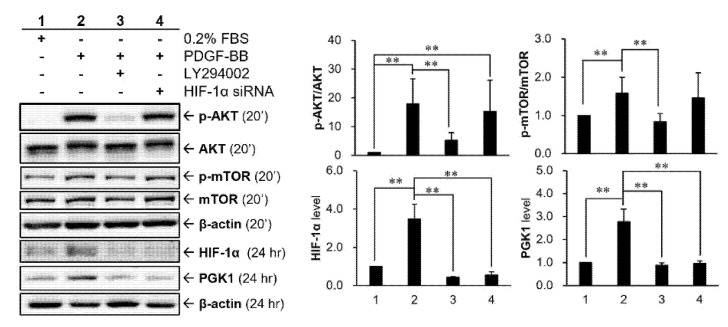
Role of HIF-1α in PDGF-BB-mediated PGK1 expression in VSMCs. Cells were pre-transfected with siRNAs for 48 h and then treated with 25 ng/mL PDGF-BB for 20 min or 24 h. Confirmation of the relationship between PDGF receptor-β (PDGFRB)-mediated signaling cascade and PGK1 expression. Cells were pretreated with 30 μM LY294002, a PI3K inhibitor, for 30 min (or pretreated with HIF-1 siRNA for 48 h) and then co-treated with 25 ng/mL PDGF-BB for 20 min or 24 h. siPDGFRB, PDGFRB siRNA; siNC, negative control siRNA. Data are representative of three independent experiments. The significance level is denoted with ** for *p* < 0.01.

For clarifying whether there is an interaction between HIF-1α and PI3K-AKT cascade, HIF-1α siRNA or PI3K inhibitor, LY294002, were applied to VSMCs (Figure 6). Our data showed that the PI3K-AKT-mTOR pathway was activated in VSMCs after PDGF-BB stimulation, whose effect can be significantly suppressed by PI3K inhibitor. Besides, HIF-1α protein level can also be upregulated by PDGF-BB and reduced after adding PI3K inhibitor, indicating that the expression of HIF-1α protein could be regulated by the PDGF-BB-mediated PI3K-AKT-mTOR pathway. This relationship further confirmed that the intervention of HIF-1α siRNA does not alter AKT phosphorylation.

## 3. Discussion

In the present study, we attempt to understand the expression profile of PGK1 protein over the progression of neointimal formation using a rat carotid artery balloon injury model. Transient arterial occlusion induced by catheter balloon inflation creates a hypoxic condition, which might upregulate PGK1 expression at early stage of neointimal formation. Thereby, the observation at the early stage after balloon inflation is essential, and can help us to clarify the temporal expression of PGK1 protein. In addition, the neointimal layer of the injured artery was gradually formed from the seventh postoperative day and dramatically increased at 14 days after surgery. Accordingly, the critical time points were selected from early (0 h, 2 h, and 2 days), mid (7 days), and late (14 days) stages after angioplasty to examine the PGK1 level and neointimal changes in the present animal study. Our study found and demonstrated for the first time that PGK1 protein can be rapidly upregulated at early stage of restenosis progression, and silencing PGK1 can be an effective strategy to significantly reduce balloon angioplasty-induced neointima formation. A complementary DNA (cDNA) microarray-based study identified numerous genes with differential expression between injured and normal rat carotid arteries at 4, 7, and 14 days post-angioplasty using the same animal model [20]. According to that reported data, PGK1 gene was significantly upregulated at 4 days post-angioplasty, and the level of HIF-1α transcript was obviously elevated at 4 days and then declined gradually until 14 days post-surgery. However, it is unknown whether the expression level of PGK1 gene can still be detected at later time points (7 and 14 days post-angioplasty) based on the disclosure data. In the present study, PGK1 and HIF-1α proteins were significantly elevated at a very early stage (2 h post-angioplasty) of neointima formation (Figure 1), implying transcriptional levels of PGK1 and HIF-1α could be regulated less than 2 h post-injury. On the other hand, VSMC proliferation was initiated at the very early stage (<1~2 days post-surgery) of neointima formation in a rat model of balloon angioplasty [4], and it is believed that early therapeutic intervention can provide more effectively prevention in neointima formation. Our data indicated that, even if PGK1 protein was just temporarily upregulated at the very early stage of neointima formation, silencing PGK1 is still an effectively therapeutic strategy to preventing the development of neointimal hyperplasia (Figure 4).

The present study found an unexpected and interesting result that PDGF-BB can induce VEGFR1 activation (phosphorylation) in VSMCs (Figure 5A). VEGFR and PDGFR have a similar intracellular structure in the tyrosine-kinase domain, implying a possibility of agonist or partial agonist acting via cross-family interaction [21,22,23]. According to the findings from Mamer and his colleagues, the computational prediction and analysis of surface plasmon resonance suggested that there is a cross-family interaction between PDGF and VEGFR [24]. Similarly, it has also been evidenced that VEGF can stimulate PDGF receptors to induce cell migration and proliferation of human adult mesenchymal stem cells [25]. Our study revealed that PDGF-BB-induced VEGFR1 activation of VSMCs is a PDGFRB-dependent regulation rather than a direct interaction between PDGF-BB and VEGFR1 (Figure 5C). In addition, our data suggested that activated VEGFR1 may also contribute partial modulations on AKT activation (Figure 5C). A previous study also showed a similar regulation that VEGF-A can stimulate cell proliferation and migration in human umbilical vein endothelial cells via activating PI3K protein, an upstream kinase of AKT protein [26]. Besides, we also evaluated whether VEGFR2 was involved in PDGF-BB-mediated PGK1 expression. Our results showed that VEGFR2 transcript was not detected in VSMCs (Figure S1 in the Supplementary information).

Systemic hypoxemia is a common finding in patients undergoing cardiac catheterization or angioplasty [27]. HIF-1α, a well-known hypoxia marker, can be rapidly and largely upregulated as a transcription factor to participate in hypoxia-mediated gene expressions or cellular responses [28]. According to the study from Karshovska et al., HIF-1α mRNA began to increase in injured carotid arteries at 2 h post-injury in a wire-induced injury model of apolipoprotein E deficient mice, and its translational level can be detected continuously at 2 weeks post-injury [8]. Our result showed a similar tendency of HIF-1α until 14 days post-injury (Figure 1). Therefore, even if HIF-1α was activated at a very early stage, its sustained expression can still affect the progression of vascular remodeling 2 weeks later [8]. Numerous glycolytic enzymes, including PGK1, have been shown to be up-regulated by HIF-1α to facilitate the catabolism and energy production required for cell survival and proliferation [10,29]. PGK1 has been indicated to promote cellular invasiveness (or dissemination), and its overexpression displayed a higher proliferative index and the capability to contribute to cell invasion [30]. The above functions of PGK1 appear to be involved in the activation of the C-X-C motif ligand-12 (CXCL12)-CXCL receptor-4 (CXCR4) signaling and its downstream molecules, such as matrix metalloproteinase-2 (MMP-2), MMP-3, AKT, and extracellular regulated protein kinases-1/2 (ERK1/2) [31]. PGK1 can also act as a protein kinase to phosphorylate pyruvate dehydrogenase kinase 1 (PDHK1), whose regulation coordinates glycolysis and the tricarboxylic acid (TCA) cycle in cancer metabolism and tumorigenesis [32]. In addition to the function in glycolysis, PGK1 has also been identified as a primer recognition protein to act as a cofactor of DNA polymerase alpha and may have a role in lagging strand DNA replication [33].

HIF-1 and PDGF have been reported to have complex relationship, including that both proteins can inter-regulate each other and that the cotreatment shows a synergistic effect. In human cancer cells, HIF-1α can upregulate the level of PDGF transcript [17,34]. It has also been reported that reactive oxygen species-stimulated HIF-1α played a critical role involved in PDGF-BB induction mediated by human immunodeficiency virus (HIV)-protein [35]. In contrast to the regulation of HIF-1α on PDGF, it has also been demonstrated that PDGF-BB treatment can rapidly increase the nucleus level of HIF-1 to activate transcription of myeloid cell leukemia-1 (Mcl-1) in human prostate cancer cells [36]. On the other hand, HIF-1α has a synergistic effect with PDGF to stimulate VSMC proliferation under hypoxia [37]. The research from Lau and his colleagues reported an inter-relationship that AKT/HIF-1α/PDGF-BB autocrine loop mediated hypoxia-induced chemoresistance in cancer cells [38]. The above studies indicated that hypoxia induced HIF-1α expression as well as AKT activation, and suppressing AKT activation will result in a significant decrease of HIF-1α expression under hypoxic conditions. Further, their data also found that exogenous PDGF-BB recombinant protein can stimulate AKT activation. Thereby, HIF-1α-mediated increase of PDGF-BB production may be due to an autocrine loop that activated AKT, thus linking the AKT and HIF-1α pathways. Our data suggested that PDGF-stimulated PGK1 expressions are regulated via HIF-1α, because inhibiting HIF-1α resulted in a significant reduction of PGK1 expression (Figure 6). In addition, HIF-1α seems to augment the expression level of PGK1 protein induced by PDGF, although this regulation has no statistical difference (Figure 5B).

AMPK is a critical intracellular sensor for energy homeostasis, and its activity can be positively regulated by numerous factors, such as exercise, hormones, dietary restriction, and energy deficiency (increases in cytosolic AMP or ADP) [39,40]. A previous article evidenced that suppressing glycolysis by an inhibitor (2-deoxy-d-glucose) of glucose metabolism can induce AMPK activation, which may be owing to the imbalance of intracellular ATP production and depletion [41]. The mTOR kinase is a central controller of cell growth and protein synthesis [42,43], and AMPK has two different regulatory mechanisms to suppress mTOR kinase by influencing mTOR complex 1 (mTORC1). The first mechanism is by activating tuberous sclerosis complex-2 (TSC2) to suppress Rheb (Ras homolog enriched in brain), which can bind with mTORC1 to activate mTOR kinase [44,45]. The second one is by deactivating (phosphorylating) regulatory-associated protein of mTOR (Raptor), a subunit of mTORC1, to inhibit mTOR kinase [46]. In addition to the regulation on mTOR, AMPK is also able to induce the activation (phosphorylation) of p53, a tumor suppressor gene, to initiate cell-cycle arrest or apoptotic cell death [47,48]. Additionally, AMPK can activate p53 via inhibiting p53 ubiquitylation and subsequent degradation [49]. In our study, activated AMPK was increased in VSMCs under PGK1 knockdown-induced energy depletion (Figure 3). Silencing PGK1-caused growth inhibition may attribute to AMPK-mediated inhibition in cell-cycle (G1 phase arrest) and induction in programmed cell death (sub-G1 phase) (Figure 2C).

A previous report used an in vivo siRNA delivery system to silence gene expression in the same animal model as ours, and their data showed that siRNA that dissolved in pluronic gel and then applied on the outside (adventitia layer) of carotid arteries can diminish endogenous expression of VEGF-A by approximately 40% at 48 h [50]. Our study, using the same experimental procedure, showed that this in vivo siRNA intervention can evidently reduce PGK1 expression at 2 h post-injury (Figure 4C) as compared with the original expression profile of PGK1 protein in the injured arteries (Figure 1). In the present study, in vivo transfection of siPGK1 can indeed achieve a significant inhibition in neointima hyperplasia at 14 days post-surgery (Figure 4B).

Our data from an animal study indicated that blocking PGK1 expression can be an effective strategy to reduce neointimal formation, although PGK1 protein only appeared briefly at very early stage after surgical intervention with a balloon catheter (Figure 1 and Figure 4). These data suggested that PGK1 not only acted as an early response gene participated in neointimal formation, but has also great potential as a therapeutic target for preventing restenotic progression. In addition, a short-term intervention targeting early response genes (e.g., PGK1) could also provide a similar efficacy at preventing restenosis as long-term drug release. To date, numerous PGK1 blockers, such as CBR-470-1, NG52, and GQQ-792, have been developed as potential anti-cancer drugs with inhibitory mechanisms on tumor growth, metastasis, the epithelial–mesenchymal transition process, as well as chemotherapy resistance [51,52,53]. However, it is largely unknown whether these PGK1 inhibitors or their derivatives can be applied for preventing neointimal formation. Thereby, functional and safety analyses of these PGK1 inhibitors or their derivatives at preventing neointimal formation are needed in future.

**Figure 7:**
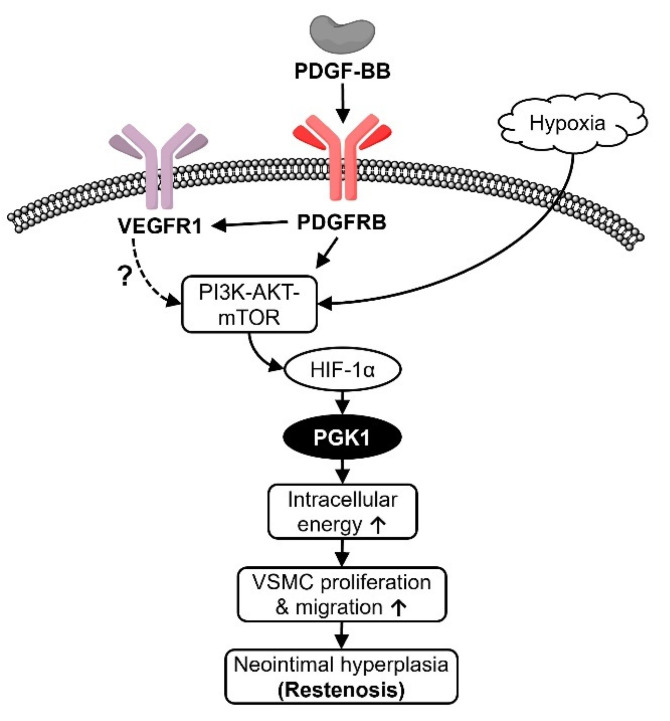
Schematic overview of upstream regulation of PGK1 and its role in neointimal hyperplasia. HIF-1α, hypoxia-inducible factor-1α; mTOR, mechanistic target of rapamycin kinase; PDGF-BB, platelet-derived growth factor-BB; PDGFRB, PDGF receptor-β; PGK1, phosphoglycerate kinase 1; PI3K, phosphoinositide 3-kinases; VEGFR1, VEGF receptor-1; VSMC, vascular smooth muscle cells.

In conclusion, PGK1 protein was rapidly upregulated by hypoxia at the very early stage of neointima formation in the rat model. In vitro knockdown of PGK1 gene showed the impact on VSMC proliferation and migration, whose findings provided some regulatory mechanisms to support in vivo data that silencing PGK1 can effectively prevent restenosis formation. Based on the previous studies and our findings, we delineated a possible mechanism that PDGF may coordinate with hypoxia to stimulate PGK1 expression by PI3K-AKT-mTOR-HIF-1α signaling cascade, and then result in neointima formation of balloon-injured arteries (Figure 7).

## 4. Materials and Methods

### 4.1. Chemicals

The primary antibodies against AMPKα (#2793s), HIF-1α (#3716), phospho-AMPKα (#2535s), and mTOR (#2972s) were purchased from Cell Signaling Technology (Beverly, MA, USA). The antibodies targeting phospho-AKT (#ab28821), PGK1 (#ab38007), and phospho-mTOR (#ab109268) were obtained from Abcam (Cambridge, MA, USA). The primary antibodies against β-actin (#GTX109639), PDGFRB (#GTX61115), phospho-PDGFRB (#GTX61797), phospho-VEGFR1 (#GTX32184), and VEGF (#GTX61100) were purchased from GeneTex (Irvine, CA, USA). Horseradish peroxidase (HRP)-conjugated secondary antibodies against mouse (#GTX213112-01) and rabbit (#GTX213110-01) immunoglobulin G (IgG) were also bought from GeneTex. LY294002 was obtained from Cayman Chemical (Ann Arbor, MI, USA). Recombinant proteins of VEGF-A (#400-31) and PDGF-BB (#100-14B) were bought from PeproTech (Rehovot, Israel). All other reagents that are not listed were purchased from Sigma-Aldrich (St. Louis, MO, USA).

### 4.2. Cell Culture

VSMCs of rat thoracic aorta (A10 cells; #BCRC-60127) were purchased from Food Research and Development Institute (Hsinchu, Taiwan) and cultured in Dulbecco’s modified Eagle’s medium (DMEM, # 12800-017; Gibco™, Thermo Fisher Scientific, Waltham, MA, USA) supplemented with 10% FBS (#35-010-CV; Corning, Glendale, AZ, USA) and a 100×-diluted antibiotic antimycotic solution (#SV30079.01; HyClone™, Cytiva, Marlborough, MA, USA). Cells were kept at 37 °C in a humidified incubator with 5% (*v*/*v*) CO_2_ and 95% (*v*/*v*) air. The culture medium was refreshed every 2~3 days.

### 4.3. Transfection of Specific siRNA

The siRNA oligonucleotides (Table 1) of PGK1, HIF-1α, and PDGFRB genes as well as negative control (scramble siRNA) were synthesized by Shanghai GenePharma Co., Ltd. (Shanghai, China). Transfection procedure was performed according to the manufacturer’s instructions (Mirus Bio, Madison, WI, USA). In brief, about 70% confluent cells were seeded on wells of a six-well plate for 24 h. On the day of transfection, 10 μL of TransIT-TKO^®^ transfection reagent (#MIR-2150; Mirus Bio) was mixed thoroughly with 250 μL of serum-free medium and then incubated at room temperature for 20 min. The siRNA oligonucleotide was gently mixed with diluted transfection reagent to achieve a final concentration of 30 nM and incubated at room temperature for 20 min for complex formation. In the meantime, the cell media were replaced with 1.25 mL of fresh complete growth medium. The siRNA/transfection reagent complexes were added drop-wise into the wells with A10 cells, and the plate was gently rocked back and forth and from side to side to distribute the complexes evenly. The silencing efficiencies were examined by immunoblot after 48 h of siRNA transfection.

For in vivo siRNA study, the preparation and procedure of siRNAs transfection were carried out according to the manufacturer’s instructions (Polyplus-transfection, New York, NY, USA) and previous study [50]. First, 80 μg of siRNA (in TE buffer containing 10 mM Tris-HCl, pH = 7.5, and 1 mM EDTA) and 16 μL of in vivo-jetPEI reagent (#201-100G; Polyplus-transfection) were diluted in 25 μL of 10% (*w*/*v*) glucose stock solution, respectively. After 15 min incubation at room temperature, the mixture of siRNA/in vivo-jetPEI reagent was further mixed well in 150 μL of 40% (*w*/*v*) pluronic F-127 (Sigma-Aldrich) with 5% (*w*/*v*) glucose (N/P ratio = 10, which is defined as the number of nitrogen residues [N] of in vivo-jetPEI reagent per nucleic acid phosphate [P]). The adventitial surface of the injured arteries was surrounded by 200 μL of pluronic gel with siRNA/transfection reagent complex. Two weeks after balloon-injury angioplasty, the animals were sacrificed and common carotid arteries were harvested for further histological examination, as described above. The siRNA silencing efficiency was confirmed using Western blot to examine the protein expression within the injured arteries.

### 4.4. Cell Viability Assay (MTT Assay)

Cells were seeded on 24-well plates (5 × 10^4^ cells/well) overnight, and then cells were transfected with 30 nM siRNA oligonucleotide in a total volume of 300 μL of culture medium with 10% FBS for 24 h. After 24 h of incubation, 10 μL of 5 mg/mL of MTT (3-[4,5-dimethylthiazol- 2-yl]-2,5-diphenyl tetrazolium bromide) was added into each well. After 3 h of incubation, the culture media were removed and then 100 μL of dimethyl sulfoxide was added to each well. Absorbance was measured at 570 nm and corrected for background absorbance using a wavelength of 650 nm. The relative absorbance is proportional to the percentage of viable cells.

### 4.5. Cell Migration Assay

The VSMCs’ migration was evaluated using Oris^®^ cell migration assay kit (#CMA1.101) following the manufacturer’s instruction by Platypus Technologies (Madison, WI, USA). Briefly, at 24 h after siRNA (30 nM) transfection, VSMCs were trypsinized and resuspended in culture medium at 2 × 10^5^ cells/mL and the cell suspension (100 μL) was added to each well of a 96-well plate plugged with a seeding stopper. After overnight adaptation and a further 24 h of starvation at 37 °C in a 5% CO_2_ incubator, the seeding stoppers were carefully pulled out from the plate well, and each well was gently washed with culture medium. Attached cells were incubated with complete culture medium with 10% FBS (as migration stimulator) for 18 h. For visualizing the cellular morphology, cells were fixed with methanol for 15 min and then stained with 10-fold diluted Giemsa stain solution (#GS500; Sigma-Aldrich) for 30 min. The image was acquired under microscopy after the plate mounted with a detection mask, and the cells migrated into the detection zone were counted using ImageJ^®^ software (National Institute of Health, Bethesda, MD, USA). Cell number of the migrated cells in the group treated with 10% FBS + NTs was defined as 100%.

### 4.6. Cell Cycle Analysis

The treated cells were harvested, washed twice with ice-cold 1× phosphate buffered saline (PBS), and then fixed with 70% ice-cold ethanol overnight. The fixed cells were centrifuged (800× *g*, 5 min, 4 °C), washed twice using 1× PBS with 0.5% (*w*/*v*) bovine serum albumin (BSA), and then re-suspended in 500 μL of DNA staining buffer containing 4 μg/mL propidium iodide, 1% (*v*/*v*) Triton X-100, and 0.5 mg/mL of RNase A. After incubation at 37 °C for 30 min in the dark, the cell cycle phase distributions were detected and analyzed using FACSCanto flow cytometer (BD Biosciences, San Jose, CA, USA) and ModFit LT Program (Verify Software House, Topsham, ME, USA), respectively.

### 4.7. Measurement of Cellular Energy Status

The intracellular ADP/ATP ratio was used to evaluate cellular energy status. The experiment procedure was performed according to the manufacturer’s instruction of EnzyLight™ ADP/ATP Ratio Assay Kit (#ELDT-100; BioAssay Systems, Hayward, CA, USA). In brief, the cells seeded on a 96-well plate (600 cells/well) were transfected with 30 nM of PGK1 or scramble siRNAs for 72 h. After that, culture medium was removed and 90 μL ATP reagent (mixed by the ratio of 95 μL assay buffer, 1 μL substrate, 1 μL cosubstrate, and 1 μL ATP enzyme) was added into each well. One minute later, the luminescent signals (RLU_1_) of samples were detected using a luminometer (Varioskan Flash™; Thermo Scientific). After waiting for 10 min, the luminescent signals (RLU_2_) were read once. Subsequently, 5 μL of ADP reagent (mixed by the ratio of 5 μL H_2_O and 1 μL ADP enzyme) was added into each well. After incubation for 1 min, the luminescent signals (RLU_3_) of the samples were measured again. Finally, the intracellular ADP/ATP ratio of each sample was calculated using the equation below.

ADP/ATP Ratio = (RLU_3_ − RLU_2_)/RLU_1_

### 4.8. Induction of Hypoxic Condition (Anaerobic Cultivation)

An anaerobic atmosphere (<0.1% oxygen concentration) of cell culture was conducted by the method of the AnaeroPack^®^-Anaero system (Mitsubishi Gas Chemical; Tokyo, Japan). In brief, cells seeded on a culture plate were placed into a sealed anaerobic jar with an oxygen-absorber sachet. The anaerobic jar was then incubated for 24 h in a CO_2_ incubator at 37 °C.

### 4.9. Rat Carotid Artery Balloon Angioplasty

Male Sprague-Dawley rats (200–350 g, 12-week-old) were purchased from BioLASCO (Taipei, Taiwan) and housed under 12 h light/dark cycles and fed ad libitum in the Laboratory Animal Service Center, China Medical University (CMU). All procedures and animal care were approved (approval number: 100-49-N and date of approval: 15 December 2010) by the Institutional Animal Care and Use Committee (IACUC) of CMU and all animal care followed the institutional animal ethical guidelines of CMU. SD rats were anesthetized by intraperitoneal injection with 20 mg/kg of Zoletil 50^®^ (Virbac, Carros, France) and 5 mg/kg of Rompun^®^ (Bayer; Mississauga, ON, Canada), and balloon angioplasty-induced neointimal hyperplasia was induced by an arterial embolectomy catheter (2F × 80 cm; Biosensors International Technology, Jalan Tukang, Singapore) in the left common carotid artery. Animals were sacrificed at 0 h, 2 h, 2 days, 7 days, and 14 days post-angioplasty, respectively (*n* = 3 animals/time point). Animals were transcardially perfused with normal saline until the perfusate was free of blood. After that, 10% formalin perfusion fixation was carried out if the arterial tissues will be applied for histological examination. For immunoblot analysis, the fresh arteries were immediately frozen and stored at −80 °C for further processing. Both (right and left) common carotid arteries were collected. For histological examination, the harvested arterial tissues were embedded in paraffin, crosscut into 10 μm thick sections, and then stained by the method of hematoxylin-eosin Y (Merck, Kenilworth, NJ, USA). The areas of the (neo)intimal and media layers of the arterial wall were measured with Image J software. The neointimal layer was defined as the tissue between the lumen surface and the internal elastic fiber, and the medial layer was defined as the tissue between the internal and external elastic fibers. The (neo)intima-to-media area ratio (I/M ratio) was used to evaluate the extent of neointimal hyperplasia.

### 4.10. Protein Extraction and Sample Preparation

Total protein extraction of fresh arteries was performed using a homogenizer in PRO-PREP extraction solution (#17081; iNtRON Biotechnology, Gyeonggi-do, South Korea) and the supernatants were collected by centrifuged at 14,000× *g* for 10 min at 4 °C. For extraction of cellular proteins, the treated cells were directly lysed using same lysis solution and centrifuged at 14,000× *g* at 4 °C for 10 min to collect the supernatants. All supernatants were collected and stored at −80 °C for further use. Protein concentrations of harvested supernatants were measured by using the Bio-Rad protein assay kit (Bio-Rad; Hercules, CA, USA) according to the manufacturer’s instructions and using BSA serial dilution to develop standard curve.

### 4.11. Immunoblot

Aliquots containing 30~50 μg total proteins were separated by 10% SDS-PAGE. After electrophoresis, the proteins in the gels were electrophoretically transferred to polyvinylidene difluoride (PVDF) membranes (Immobilon-P; Millipore, Temecula, CA, USA). After blocking non-specific binding sites by 5% (*w*/*v*) non-fat milk, the membrane was processed for reactions with primary and secondary antibodies. Chemiluminescent signals were developed with the LumiFlash Ultima Chemiluminescent substrate (#VPLF08-500; VisualProtein, Taipei, Taiwan) and then acquired by the Azure C300 imaging System (Azure biosystem, Dublin, CA, USA) with default settings (sensitivity: normal; exposure time: auto expose). The band intensities of identified proteins were normalized to that of internal control protein (β-actin), and the expression levels of identified proteins were presented as fold change to the value of the control group.

### 4.12. Statistical Analysis

SPSS Statistics software (vers. 19; IBM, Armonk, NY, USA) was used to perform the statistical analysis. All values were presented as mean ± standard deviations (SD). An unpaired t-test was used to analyze the significant difference between two siRNA intervention groups in cell viability assay. In multiple-group experiments, a one-way analysis of variance (ANOVA) combined with a post hoc test (Dunnett’s test) was used to determine significant differences. A *p*-value less than 0.05 was considered statistically significant. The significance level is denoted by * and † for *p* < 0.05, and ** and ‡ for *p* < 0.01.

## Figures and Tables

**Table 1:** The sequences of siRNA oligonucleotides.

Target Genes (Accession No.)	siRNA Sequences (5′-3′)
HIF-1α (NM_024359)	GGAAAGAGAGUCAUAGAAATT (sense) UUUCUAUGACUCUCUUUCCTT (antisense)
PDGFRB (NM_031525)	GGUGGUGUUUGAGGCUUAUTT (sense) AUAAGCCUCAAACACCACCTT (antisense)
PGK1 (NM_053291)	GUACUGAGAGCAGUAAGAATT (sense) UUCUUACUGCUCUCAGUACTT (antisense)
Negative control	UUCUCCGAACGUGUCACGUTT (sense) ACGUGACACGUUCGGAGAATT (antisense)

HIF-1α, hypoxia-inducible factor-1α; PDGFRB, PDGF receptor-β; PGK1, phosphoglycerate kinase 1.

## Data Availability

The data used to support the findings of this study are available from the corresponding author upon reasonable request.

## Supplementary Materials

The following are available online at https://www.mdpi.com/article/10.3390/ijms22168822/s1.
