# Supplementary material for: Pathological Role of Phosphoglycerate Kinase 1 in Balloon Angioplasty-Induced Neointima Formation
Source: Int J Mol Sci. 2021 Aug 17;22(16):8822. doi: 10.3390/ijms22168822 (PMC8396187; doi:10.3390/ijms22168822)
Supplement: Supplementary file 1 [file ijms-22-08822-s001.zip › ijms-1330194-supplementary.pdf]

The supplementary Material for

Pathological Role of Phosphoglycerate Kinase 1 in Balloon Angioplasty-Induced Neointima Formation

Chun-Hsu Pan[1,2], Yi-Chung Chien[3,4], Min-Shan Sung[5], Hui-Yu Huang[6], Ming-Jyh Sheu[7], Chieh-Hsi Wu[2,*]

[1] Ph.D. Program in Drug Discovery and Development Industry, College of Pharmacy, Taipei Medical University

[2] School of Pharmacy, Taipei Medical University, Taipei 11031, Taiwan

[3] Drug Development Center, Research Center for Cancer Biology, China Medical University, Taichung 40402, Taiwan

[4] Center for Molecular Medicine, China Medical University Hospital, Taichung 404332, Taiwan

[5] Graduate Institute of Basic Medical Science, China Medical University, Taichung 40402, Taiwan

[6] Graduate Institute of Metabolism and Obesity Sciences, Taipei Medical University, Taipei 11031, Taiwan

[7] School of Pharmacy, China Medical University, Taichung 40402, Taiwan

***Corresponding author:**

Chieh-Hsi Wu, Ph.D.

Professor and Vice President

School of Pharmacy, Taipei Medical University

No. 250, WuXing Street, Taipei 11031, Taiwan

Tel: +886-2-27361661 ext.2047

E-mail: chhswu@tmu.edu.tw

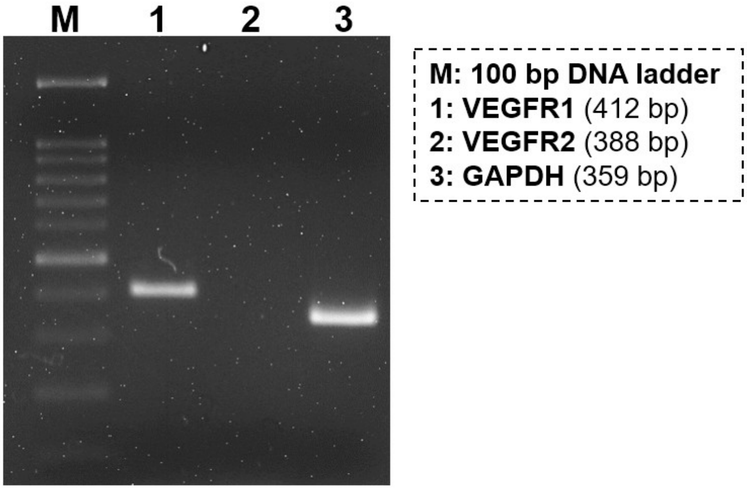

Figure S1. Detections of VEGFR1 and VEGFR2 transcripts in VSMCs by RT-PCR. Gene expression of VEGFR1 and VEGFR2 were examined using a ReverTra Ace set (TOYOBO) and an AptaTaq Fast PCR Master kit (Roche), respectively. Primer pairs were following: VEGFR1 (5'-ACAGGACCATGCACCATAGC-3'; 5'-GTGGCTCGGCACCTATAGAC-3'), VEGFR2 (5'-TCTGGACTCTCCCTGCCTAC-3'; 5'-ACACGGTGGTATCTGTGTCG-3'), and GAPDH (5'-ATCAAGAAGGTGGTGAAGCAGGCG-3'; 5'-GGGATGGAATTGTGAGGGAGATGCTC-3’).
